# Sexual behaviour does not reflect HIV-1 prevalence differences: a comparison study of Zimbabwe and Tanzania

**DOI:** 10.1186/1758-2652-13-45

**Published:** 2010-11-16

**Authors:** Munyaradzi P Mapingure, Sia Msuya, Nyaradzai E Kurewa, Marshal W Munjoma, Noel Sam, Mike Z Chirenje, Simbarashe Rusakaniko, Letten F Saugstad, Sake J de Vlas, Babill Stray-Pedersen

**Affiliations:** 1 Department of Community Medicine, University of Zimbabwe, Harare, Zimbabwe; 2 Division of Obstetrics and Gynaecology, Faculty of Medicine, University of Oslo and Rikshospitalet, Oslo, Norway; 3 Department of Public Health, Erasmus MC, Rotterdam, The Netherlands; 4 Kilimanjaro Christian Medical Centre, Moshi, Tanzania; 5 Department of Obstetrics and Gynaecology, University of Zimbabwe, Harare, Zimbabwe; 6 Letten Research Centre, University of Oslo, Oslo, Norway

## Abstract

**Background:**

Substantial heterogeneity in HIV prevalence has been observed within sub-Saharan Africa. It is not clear which factors can explain these differences. Our aim was to identify risk factors that could explain the large differences in HIV-1 prevalence among pregnant women in Harare, Zimbabwe, and Moshi, Tanzania.

**Methods:**

Cross-sectional data from a two-centre study that enrolled pregnant women in Harare (N = 691) and Moshi (N = 2654) was used. Consenting women were interviewed about their socio-demographic background and sexual behaviour, and tested for presence of sexually transmitted infections and reproductive tract infections. Prevalence distribution of risk factors for HIV acquisition and spread were compared between the two areas.

**Results:**

The prevalence of HIV-1 among pregnant women was 26% in Zimbabwe and 7% in Tanzania. The HIV prevalence in both countries rises constantly with age up to the 25-30 year age group. After that, it continues to rise among Zimbabwean women, while it drops for Tanzanian women. Risky sexual behaviour was more prominent among Tanzanians than Zimbabweans. Mobility and such infections as HSV-2, trichomoniasis and bacterial vaginosis were more prevalent among Zimbabweans than Tanzanians. Reported male partner circumcision rates between the two countries were widely different, but the effect of male circumcision on HIV prevalence was not apparent within the populations.

**Conclusions:**

The higher HIV-1 prevalence among pregnant women in Zimbabwe compared with Tanzania cannot be explained by differences in risky sexual behaviour: all risk factors tested for in our study were higher for Tanzania than Zimbabwe. Non-sexual transmission of HIV might have played an important role in variation of HIV prevalence. Male circumcision rates and mobility could contribute to the rate and extent of spread of HIV in the two countries.

## Background

There is substantial heterogeneity in HIV-1 prevalence within sub-Saharan Africa, a region that contains more than a third of the world's HIV-1 infections [1]. Sub-Saharan Africa's epidemics vary significantly from country to country in both scale and scope. Adult national HIV prevalence is less than 2% in countries of west and central Africa, and in 2007, it exceeded 15% in southern African countries [2].

Zimbabwe and Tanzania are examples of sub-Saharan countries that show large variations in HIV prevalence. Zimbabwe is severely affected by the HIV and AIDS epidemic. The country is experiencing a decline in HIV prevalence, but the figures are still very high. Among pregnant women (15-49 years), HIV prevalence declined from 32% in 2000 to 26% in 2002 and 18% in 2006 [3]. In the general population, HIV prevalence in Zimbabwe was estimated to be 27% in 2001, 19% in 2005, 16% in 2007 [3] and 14% in 2009 [4]. The prevalence of the infection in Tanzania is relatively low when compared with that of Zimbabwe, and was estimated to be 12% in 1999 and 7% in 2003/04 [5]. The HIV prevalence rate among Tanzanian antenatal clinic attendees in 2005/06 was 8%, and in 2008, it was estimated to be 6% [6].

A lot of resources have been invested to identify plausible risk factors of HIV that may explain why certain areas experience very high HIV-1 prevalences [7-9]. A number of biologic, behavioural and demographic factors have been suggested as influences on the large differences in HIV prevalence in sub-Saharan Africa. These include patterns of sexual networking, other sexually transmitted infections (STIs), reproductive tract infections (RTIs), time of introduction of the virus into the general population, migration, mobility, individual differences in susceptibility to HIV, virus subtypes and male circumcision rates [8,10-12]. However, to date, there are still questions and not answers about what might be fuelling the epidemic in some countries and not in others. Comparisons of factors that determine the rate of spread of HIV in different regions is hampered by lack of comparable data [7].

A clear understanding and explanation of the striking HIV-1 differences may aid in identification of effective intervention strategies. A previous study of about 800 women in Zimbabwe and Tanzania found significant differences in HIV prevalence and called for more research to find factors that accelerate the rate of HIV acquisition or contribute to the difference in prevalence patterns [9].

This paper makes use of data from a large two-centre study done in Harare, Zimbabwe, and Moshi, Tanzania. The data were collected using the same protocol by members of the study group called Better Health for the African Mother and Child. We present here a comparison of the distribution of risk factors of HIV acquisition between the two countries. The objectives of this study are to compare underlying socio-demographic characteristics, sexual behaviour and other STIs and/or RTIs among pregnant women in Zimbabwe and Tanzania, and come up with possible explanations for the contrasting HIV-1 prevalence.

## Methods

### Study area and population

Methodology of the two centre study has been described in detail elsewhere [13,14]. Data from cross-sectional studies of pregnant women enrolled consecutively at 36 weeks of gestation between 2002 and 2004 were used; these women were enrolled at two antenatal clinics in peri-urban Moshi in Tanzania, where there is a relatively low HIV prevalence, and three antenatal clinics in the peri-urban parts of Harare in Zimbabwe, where there is a high HIV prevalence. The same protocol was used in both centres. A questionnaire was administered by interviewers to solicit information on socio-demographic background, sexual behaviour, and current and past medical history. A doctor or a midwife carried out an overall physical and gynecological examination of the women. The women were tested for HIV-1, syphilis, HSV2, *Trichomonas vaginalis*, bacterial vaginosis and candidiasis.

### Statistical analysis

Data were entered and analyzed using STATA Version 10 from StataCorp, Texas, USA. Distribution of risk factors for HIV infection between the two countries were compared using Student's t test for continuous variables and Pearson-chi square test for categorical variables. Unadjusted odds ratios and their 95% confidence intervals were presented for the various risk factors of HIV seropositivity for each country. Promising factors, i.e., those with a p value of less than 0.25 in univariate analysis, were investigated in multivariate analysis. Factors with a p < 0.10 were maintained in the final multivariate model, using a stepwise backward likelihood ratio procedure.

### Ethical approval

The studies were approved by the Medical Research Councils of the respective countries, as well as the Norwegian Ethical Committee. Every woman who consented to taking part in the study was given a numeric identifier, which was used throughout the study on all documentation to maintain patient confidentiality. The women gave written informed consent to take part in the study.

## Results

**Figure 1:**
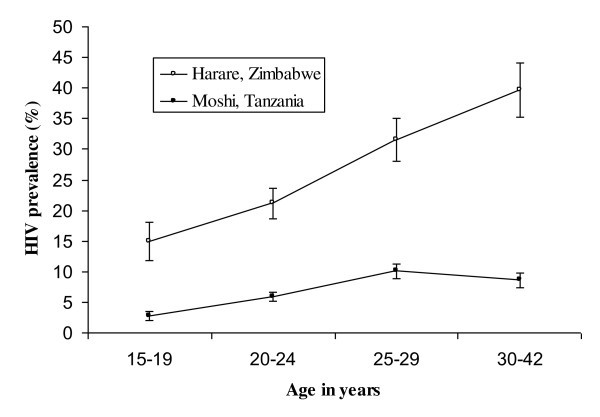
Prevalence of HIV infection by age group for 691 pregnant women in Harare, Zimbabwe and 2654 pregnant women in Moshi, Tanzania

In total, 177 (25.6%) of the 691 pregnant women in Harare, Zimbabwe and 184 (6.9%) of the 2654 pregnant women in Moshi, Tanzania were HIV-1 positive. Figure 1 compares the age-specific HIV prevalence for the two countries. HIV-1 prevalence rises with age for the two countries up to the age group of 25-29 years. Thereafter, the prevalence of HIV in Zimbabwe continues to rise while that for Tanzania drops slightly for women who are older than 30 years.

Table 1 shows a comparison of the distribution of key risk factors between the two countries. Rates of risky sexual behaviour and alcohol consumption were consistently higher in Tanzania than in Zimbabwe. Sexually transmitted infections and reproductive tract infections are more common in Zimbabwe than in Tanzania. About 92% of the male partners of the Zimbabwean women are not circumcised, while circumcision is common (98%) in Tanzania in this study. Mobility is more common in Zimbabwe (7.7% for women and 41% for their partners) than in Tanzania (1.5% for women and 26% for their partners). A history of schistosomiasis was more often reported by the women in Zimbabwe than those in Tanzania (18% vs. 4%)

**Table 1:** Comparison of HIV risk factors for 691 pregnant women (mean age 24.2 years) in Zimbabwe and 2654 pregnant women (mean age 24.6 years) in Tanzania

Variable	Harare, Zimbabwe		Moshi, Tanzania		p value
	n/N	%	n/N	%	
*HIV-positive status*	177/691	25.6	184/2654	6.9	< 0.001
*Socio-demographic characteristics and risky sexual behaviour*					
Casual sex partner in last 12 months	19/685	2.8	177/2654	4.4	0.054
More than one lifetime sexual partner	189/685	27.6	1164/2654	43.9	< 0.001
Sexual debut before 16 years	67/685	9.8	366/2654	13.8	0.005
Never used condoms	374/676	55.3	2004/2654	75.5	< 0.001
Use herbs to tighten vagina	96/685	14.0	97/1330	7.3	< 0.001
Siblings have different fathers	99/653	15.2	1437/2654	54.1	< 0.001
Polygamous relationship	54/674	8.0	296/2654	11.2	0.018
Drinks alcohol	21/677	3.1	821/2654	30.9	< 0.001
Travels outside residential area frequently	53/690	7.7	36/2413	1.5	< 0.001
*Other infections, signs and symptoms*					
HSV-2 positive	323/632	51.1	427/1271	33.6	< 0.001
Syphilis positive	8/662	1.2	23/2654	0.9	0.638
Trichomoniasis positive	80/680	11.8	127/2555	5.0	< 0.001
Bacterial vaginosis positive	195/598	32.6	533/2555	20.9	< 0.001
History of schistosomiasis	120/679	17.7	56/1332	4.2	< 0.001
Currently have genital warts	44/601	7.3	33/2599	1.3	< 0.001
Currently have genital ulcers	16/594	2.7	41/2555	1.6	0.073
Had genital warts in the last 12 months	33/686	4.8	48/2654	1.8	< 0.001
Had genital ulcer in the last 12 months	44/687	6.4	85/2654	3.2	< 0.001
*Partner characteristics*					
Current partner not circumcised	606/657	92.2	52/2413	2.1	< 0.001
Current partner travels frequently	268/659	40.7	619/2413	25.6	< 0.001

n: number HIV positive; N: number tested

Table 2 shows the risk of being HIV positive within populations, separately for data from Zimbabwe and Tanzania respectively. In both countries, several risk factors for HIV positivity were identified in the univariate analysis. In multivariate analysis for the separate countries, age, higher number of lifetime sexual partners, HSV-2 infection, bacterial vaginosis and having a genital ulcer were consistently and independently associated with HIV-1 positivity.

**Table 2:** Socio-demographic, sexual behaviour and biological risk factors for HIV infection among women in Zimbabwe and Tanzania

Determinants of HIV transmission	Harare, Zimbabwe				Moshi, Tanzania			
	Number tested	Number HIV positive (%)	Unadjusted OR^1 ^(95% CI)	Adjusted OR (95% CI)	Number tested	Number HIV positive (%)	Unadjusted OR^1 ^(95% CI)	Adjusted OR (95% CI)
**All**	691	177 (25.6)			2654	184 (6.9)		
**Socio-demographic factors**								
*Age group in years*								
< 20	134	20 (14.9)	1	1	479	13 (2.8)	1	1
20-24	265	56 (21.1)	1.5 (0.9-2.8)	2.5 (1.1-5.9)*	996	59 (5.9)	2.2 (1.2-4.1)	2.2 (1.1-4.4)*
25-29	168	53 (31.2)	2.6 (1.5-4.7)**	3.1 (1.3-7.1)*	664	67 (10.1)	4.0 (2.2-7.3)	4.4 (2.2-8.9)**
> = 30	121	48 (39.8)	3.7 (2.1-6.8)**	3.9 (1.6-9.3)*	523	45 (8.6)	3.3 (1.8-6.2)	3.2 (1.5-6.6)*
*Parity*								
No child	270	45 (16.8)	1	-	1064	51 (4.8)	1	
One ore more	419	132 (31.5)	2.3 (1.6-3.4)**	-	1590	133 (8.4)	1.8 (1.3-2.6)**	
*Marital status*								
Married/cohabiting	649	166 (25.6)	1	-	2414	161 (6.7)	1	-
Single/d/s/w^2^	40	11 (27.5)	1.1 (0.5-2.3)	-	240	23 (9.6)	1.5 (0.9-2.4)*	-
*Years in school*								
8 or more	568	144 (25.4)	1	-	271	20 (7.4)	1	-
Less than 8	123	33 (26.8)	1.1 (0.7-1.7)	-	2383	164 (6.9)	0.9 (0.6-1.6)	-
*Type of marriage*								
Monogamy	620	153 (24.7)	1	-	2358	139 (5.9)	1	1
Polygamy	54	21 (38.9)	1.9 (1.0-3.6)**	-	296	45 (15.2)	2.9 (1.9-4.1)**	1.8 (1.2-2.7)*
*Alcohol consumption*								
No	656	170 (25.9)	1	-	1833	106 (5.8)	1	-
Yes	21	3 (14.3)	0.5 (0.1-1.7)	-	821	78 (9.5)	1.7 (1.2-2.3)**	-
*Travelling*								
Rarely	637	167 (26.2)	1	-	2377	169 (7.1)	1	-
Frequently	53	10 (18.9)	0.7 (0.3-1.4)	-	36	5 (14.0)	1.4 (0.8-2.5)	-
**Sexual behaviour**								
*Sexual partners in the past 12 months*								
One only	666	167 (25.1)	1	-	2537	171 (6.7)	1	-
More than one	19	9 (47.4)	2.7 (1.1-6.7)**	-	117	13 (11.1)	1.7 (1-3.1)	-
*Lifetime sexual partners*								
One	496	93 (18.8)	1	1	1490	35 (2.4)	1	1
Two or more	189	83 (43.9)	3.4 (2.3-5.0)**	3.0 (1.8-5.1)**	1164	149 (12.8)	6.1 (4.2-9.2)**	3.9 (2.6-5.9)**
*Age (years) of sexual debut*								
At or after 16	618	157 (25.4)	1	-	2288	147 (6.4)	1	1
Below 16	67	19 (28.4)	1.2 (0.6-2.1)	-	366	37 (10.1)	1.6 (1.1-2.4)**	1.6 (1.1-2.4)*
*Ever used a condom*								
No	374	82 (21.9)	1	-	2004	121 (6.0)	1	-
Yes	302	91 (30.1)	1.5 (1.1-2.2)**	-	650	63 (9.7)	1.7 (1.2-2.3)**	-
*Uses herbs to tighten vagina*								
No	589	148 (25.1)	1	-	1233	90 (7.3)	1	-
Yes	96	29 (30.2)	1.3 (0.8-2.1)	-	97	6 (6.2)	0.8 (0.3-2.0)	-
*Siblings have different fathers*								
No	554	119 (21.5)	1	-	1217	59 (4.9)	1	1
Yes	99	52 (52.5)	4.0 (2.5-6.5)**	-	1437	125 (8.7)	1.9 (1.3-2.6)**	1.6 (1.1-2.4)*
**Other infections, signs and symptoms**								
*HSV-2*								
No	309	21 (6.8)	1	1	844	104 (12.3)	1	1
Yes	323	137 (42.4)	10.1 (6.2-16.6)**	5.3 (3.0-9.5)**	427	79 (18.5)	1.6 (1.2-2.2)**	3.1 (2.2-4.5)**
*Syphilis*								
No	654	160 (24.5)	1	-	2631	178 (6.7)	1	1
Yes	8	5 (62.5)	5.1 (1.2-21.8)**	-	23	6 (26.1)	4.9 (1.9-12.5)**	9.4 (2.4-36.2)**
*Trichomoniasis*								
No	600	140 (23.3)	1	-	2428	170 (7.0)	1	-
Yes	80	32 (40.0)	2.2 (1.3-3.6)**	-	127	13 (10.2)	1.5 (0.8-2.8)	-
*Bacterial vaginosis*								
No	403	72 (17.9)	1	1	2022	115 (5.7)	1	1
Yes	195	75 (38.5)	2.9 (1.9-4.3)**	3.0 (1.8-5.0)**	533	68 (12.8)	2.4 (1.7-3.4)**	2.2 (1.5-3.1)**
*History of schistosomiasis*								
No	559	140 (25.0)	1	-	1276	94 (7.4)	1	-
Yes	120	33 (27.5)	1.1 (0.7-1.8)	-	56	2 (3.6)	0.5 (0.1-1.8)	-
*Vaginal pH > 4.5*								
No	197	40 (20.3)	1	-	1673	99 (5.9)	1	-
Yes	415	113 (27.2)	1.6 (0.9-2.6)*	-	882	84 (9.5)	1.7 (1.2-2.3)**	-
*Clinical genital warts*								
No	557	129 (23.2)	1	1	2522	178 (7.1) 5	1	-
Yes	44	23 (52.3)	3.6 (1.9-7.1)**	3.0 (1.1-8.6)*	33	(15.2)	2.4 (0.9-6.1)*	-
*Clinical genital ulcer*								
No	578	140 (24.2)	1	1	2514	175 (7.0) 8	1	1
Yes	16	10 (62.5)	5.2 (1.9-14.6)**	3.6 (1.1-11.8)*	41	(19.5)	3.2 (1.3-6.3)*	2.7 (1.1-6.8)*
*Previous genital warts *								
No	653	163 (25.0)	1	-	2606	178 (6.8) 6	1	-
Yes	33	13 (39.4)	2.0 (1.0-4.0)*	-	48	(12.5)	1.9 (0.8-4.6)	-
*Previous genital ulcer*								
No	643	157 (24.4)	1	-	2569	174 (6.8)	1	-
Yes	44	19 (43.2)	2.4 (1.3-4.4)**	-	85	10 (11.8)	1.8 (0.9-3.6)*	-
**Partner characteristics**								
*Current partner circumcised*								
No	606	154 (25.3)	1	-	52	6 (11.5)	1	-
Yes	51	14 (27.5)	1.1 (0.5-2.2)	-	2361	168 (7.1)	0.6 (0.2-1.7)	-
*Current partner frequent traveler*								
No	391	98 (25.1)	1	-	1794	108 (6.0)	1	1
Yes	268	71 (26.5)	1.1 (0.7-1.6)	-	619	66 (10.7)	1.9 (1.3-2.6)**	1.9 (1.3-2.7)**

^1^OR stands for odds ratio, 95% confidence interval of the odds ratio are given ^2^d/s/w represents divorced/separated/widowed

* = p < 0.05, ** = p < 0.001

All factors with a p value of less than 0.25 in univariate analysis were included in multivariate analysis, and adjusted odds ratios which had a p value of less than 0.10 are included in this table.

Independent risk factors for HIV that were identified in Tanzania only were early age of sexual debut, being in a polygamous marriage, having children with different men, syphilis infection and having a partner who travels. These factors did not reach statistical significance in multivariate analysis in Zimbabwe, but were significant risk factors in univariate analysis, except for early age of sexual debut and having a partner who travels frequently. Having genital warts was independently associated with HIV infection in Zimbabwe, but this association was shown only in univariate analysis for Tanzania. Having a partner who is circumcised showed a tendency towards protection from HIV infection in Tanzania, but this was not statistically significant, while in Zimbabwe this factor showed the reverse association in univariate analysis and was also not significant in the multivariate analysis.

## Discussion

We saw significant differences in the HIV prevalence for women attending antenatal clinics in Harare, Zimbabwe (25.6%) and in Moshi, Tanzania (6.9%), consistent with earlier reports [9]. The HIV prevalence for both countries rises constantly with age, but while it continues to rise among Zimbabwean women older than 30 years, the graph for Tanzanian women tails off. Mobility and biological risk factors for HIV, such as STIs and RTIs, notably HSV-2, trichomoniasis and bacterial vaginosis, were more prominent among Zimbabweans than Tanzanians. Risky sexual behaviour and male circumcision were more prominent among Tanzanians than Zimbabweans. In both countries, age, higher number of lifetime sexual partners, HSV-2 and bacterial vaginosis infections and having a genital ulcer were consistently and independently associated with HIV-1 positivity.

An unexpected phenomenon was seen in the sexual behaviour data: women in Tanzania reported more risky sexual behaviour than women in Zimbabwe, which is opposite to what is reflected in the HIV prevalence. Prevalence of risky sexual behaviour characteristics, such as having had a casual sexual partner in the previous 12 months, having had more than one lifetime sexual partner, early sexual debut, being in a polygamous relationship and having siblings by different fathers, were all higher for Tanzania. Alcohol consumption, which increases the tendency to engage in risky sexual behaviour [15], was also more common in Tanzania than in Zimbabwe. Clearly, sexual behaviour only cannot explain the observed differences in HIV prevalence between the two countries. How then can we explain this paradox?

The data collected from 2002 to 2004 in Moshi and Harare are cross-sectional and thus describe the situation close to the time of data collection, whereas the HIV prevalence data are the result of exposure to risk factors over periods of a decade or more. During this time, the prevalence of some of the key risky sexual behaviours is likely to be reduced, particularly where epidemics are severe [8]. It is possible that at the time of data collection, sexual risk behaviour for the women in Zimbabwe was decreasing in response to the alarming prevalence that had caused so much morbidity and mortality.

A longitudinal study conducted in the Manicaland province, Zimbabwe, has shown an improvement in sexual risk behaviour, e.g., men reporting fewer casual sexual partners than before [16]. In some parts of Tanzania, meanwhile, studies have shown that sexual risk behaviour is not decreasing because people see themselves as not being at risk of HIV infection [17]. However, the results of 1999 and 2005 demographic and health surveys done in the two countries have consistently shown that risky sexual behaviour is more prominent in Tanzania than in Zimbabwe. This is in terms of having: extramarital sexual partners; higher risk sexual intercourse; higher percentages of both men and women not using condoms; and higher percentages of men who reported visiting a commercial sex worker [18-21].

Lower risk sexual behaviour in Zimbabwe than in Tanzania could also be a result of under-reporting of socially unacceptable sexual behaviour by Zimbabwean women. Differences in social desirability bias could be a major contributing factor to the quality of sexual behaviour data [22]. Discrepancy in risky sexual behaviour and HIV prevalence were, however, reported in other studies of heterogeneity in HIV prevalence in African countries in which data collection methods were highly standardized and included triangulation [23].

From the "Four Cities Study", behavioural factors found to be more common in the two high HIV prevalence cities were young age at first sexual intercourse (women), young age at first marriage and large age differences between spouses. However, high rate of partner change, sex with sex workers, concurrent partnerships, and larger age difference between non-spousal partners were not more common in the two high HIV prevalence cities [23].

Apart from age mixing i.e sexual partners with large age differences, a study by Chapman *et al *[22], which used adolescent data from Zimbabwe, South Africa and Tanzania, found that "behaviours assumed *a priori *to be higher risk were not found to be more common in populations with higher HIV prevalence. In some cases, risk behaviours were much more prevalent in lower HIV prevalence studies. For example, the lowest levels of having had sex, oldest age of debut and the lowest proportion of multiple partners were reported in Zimbabwe, although that country had the highest HIV prevalence" [22].

Prevalence of HSV-2 and trichomoniasis was moderately higher in Zimbabwe than in Tanzania, but HIV prevalence in Zimbabwe was almost four times higher than that in Tanzania. With regards to the interaction between STIs and HIV infection, there is convincing evidence that STIs substantially enhance the vulnerability of non-HIV-infected individuals and the infectiousness of HIV-infected individuals [24,25]. The prevalence of women with genital warts and genital ulcers was also higher in Zimbabwe than in Tanzania. It has been shown in several studies that the presence of sores on the genital tract facilitates entry of HIV [26,27].

However, the causes of the higher prevalences of STIs and genital symptoms in Zimbabwe, given the observed much lower degree of risk behaviour compared with women in Tanzania, remains questionable. In 1999, the prevalence of STIs among women in Moshi and Harare were reported to be similar, except for large HIV prevalence differences, again showing higher prevalence in Harare [9]. This suggests that the higher STI prevalences in Zimbabwe compared with Tanzania during the study period, 2002 to 2004, were caused by HIV prevalence differences that existed over time.

Male circumcision among regular or current sexual partners was reported by almost 98% of the women in Tanzania and by only about 8% of the Zimbabwean women. Three randomized controlled trials, in Uganda, Kenya and South Africa, have shown that male circumcision is associated with a decreased risk of acquisition of HIV infection by men [28-30]. Reviews by van Howe [31] and Weiss *et al *[32] show that male circumcision might be protective against other STIs as well.

In the Ugandan randomized controlled trial, the prevalence of self-reported symptoms of STIs was lower in the circumcised arm than in the control arm. Obviously, women in areas where male circumcision is common get an indirect advantage due to the protective effect for their partners and the corresponding lower HIV prevalence in the population. Even though the rates of circumcision match the HIV prevalences in our study, the protective effect of male circumcision is not visible in the data within each country. Data from Tanzania show an insignificant protective effect, which might be due to the small number of men who are not circumcised. In Zimbabwe, those who are circumcised might possess other risky characteristics, possibly cultural, which render the protective effect of male circumcision insignificant.

Some studies point to the role of mobility and schistosomiasis infection rates in HIV acquisition in sub-Saharan Africa [12,33,34]. In our study, mobility was more common among Zimbabwean women and their partners than among those in Tanzania. However, the individual-level analysis did not show any association of mobility and HIV infection, except for male partners of Tanzanian women. With regard to schistosomiasis infection, our study results show marked differences in the prevalence between the two countries, but this infection was not at all associated with HIV seropositivity within both countries.

Another possible explanation for the contrasting HIV epidemics could be the role played by non-sexual transmission of HIV that might have occurred more in Zimbabwe in the early years of the epidemic. Figure 1 shows that HIV prevalence in our results continues to increase for the Zimbabwean women who are 30 years and older, while the rate for women in Tanzania stabilizes or even decreases with age. These women grew up in the 1980 s, when a number of studies reported HIV-positive children with HIV-negative mothers [35-39].

Some studies challenge the conventional hypothesis that sexual transmission is responsible for more than 90% of adult HIV infections in Africa [40]. A study in Zimbabwe in the 1990 s found a 2.1% HIV prevalence among 933 women with no reported sexual experience [41]. If adults and adolescents with no sexual exposures are found to be HIV positive, this suggests that a proportion of the HIV in those who are sexually exposed also comes from non-sexual transmission [40].

It is, however, important to highlight the possible weakness of sexual behaviour surveys in failing to detect true differences in risk. Another vital point is that some variables may not be fully investigated. For example, in this study the phrase, "ever used condom", is used rather than the more useful, "condom use at last sexual encounter". Further, the data collected age of sexual debut in categories, not the actual age of debut, making it difficult to estimate the median value. The role of other factors, such as age mixing and concurrency in driving the HIV prevalence in different ways, should also be investigated.

## Conclusions

From our data and available information, we conclude that differences in sexual behaviour alone cannot explain the much higher HIV prevalence in Harare, Zimbabwe, than in Moshi, Tanzania. The large HIV prevalence differences may be a result of the fact that non-sexual transmission of HIV occurred at a relative larger scale in Zimbabwe in the early years of the epidemic. Male circumcision might be responsible for the low prevalence of STIs and HIV in Tanzania relative to Zimbabwe, but we could not confirm the role of male circumcision within the populations. More comparable sexual behaviour surveys that are capable of investigating risk factors fully and correctly in different countries are needed.

## Competing interests

Letten F Saugstad is the founder of the Letten Foundation, which sponsored the study in Zimbabwe and Tanzania. The other authors have no conflicts of interest to declare.

## Authors' contributions

MPM drafted the manuscript, analyzed data and interpreted results. SJDV contributed to drafting of the manuscript and interpretation of results. ENK, MWM, SM and NS participated in data collection. MZC supervised data collection. RS participated in data analysis. LFS participated in protocol development and interpretation of results. BSP developed the protocol, participated in drafting of the manuscript and interpretation of results. All authors read and approved the final version of the manuscript.
